# MicroRNA-29a activates a multi-component growth and invasion program in glioblastoma

**DOI:** 10.1186/s13046-019-1026-1

**Published:** 2019-01-25

**Authors:** Yun Zhao, Wei Huang, Tae-Min Kim, Yuchae Jung, Lata G. Menon, Hongyan Xing, Hongwei Li, Rona S. Carroll, Peter J. Park, Hong Wei Yang, Mark D. Johnson

**Affiliations:** 1 Department of Neurological Surgery, University of Massachusetts Medical School, Albert Sherman Center AS6-1001, 368 Plantation Street, Worcester, MA 01605 USA; 2 Department of Neurosurgery, Brigham and Women’s Hospital and Harvard Medical School, Boston, MA USA; 3 Department of Biomedical Informatics, Harvard Medical School, Boston, MA USA; 4 Department of Chemotherapy, Tumor Hospital of Guangxi Medical University, No.2, Nanning, Guangxi China; 5 Program in Neuro-Oncology, Dana Farber Cancer Institute, Boston, MA USA; 6 Department of Neurological Surgery, UMass Memorial Healthcare, University of Massachusetts Medical School, 55 Lake Avenue North, S2-855, Worcester, MA 01655 USA

**Keywords:** miR-29a, Glioblastoma, Invasion, Sox4, PTEN

## Abstract

**Background:**

Glioblastoma is a malignant brain tumor characterized by rapid growth, diffuse invasion and therapeutic resistance. We recently used microRNA expression profiles to subclassify glioblastoma into five genetically and clinically distinct subclasses, and showed that microRNAs both define and contribute to the phenotypes of these subclasses. Here we show that miR-29a activates a multi-faceted growth and invasion program that promotes glioblastoma aggressiveness.

**Methods:**

microRNA expression profiles from 197 glioblastomas were analyzed to identify the candidate miRNAs that are correlated to glioblastoma aggressiveness. The candidate miRNA, miR-29a, was further studied in vitro and in vivo.

**Results:**

Members of the miR-29 subfamily display increased expression in the two glioblastoma subclasses with the worst prognoses (astrocytic and neural). We observed that miR-29a is among the microRNAs that are most positively-correlated with PTEN copy number in glioblastoma, and that miR-29a promotes glioblastoma growth and invasion in part by targeting PTEN. In PTEN-deficient glioblastoma cells, however, miR-29a nevertheless activates AKT by downregulating the metastasis suppressor, EphB3. In addition, miR-29a robustly promotes invasion in PTEN-deficient glioblastoma cells by repressing translation of the Sox4 transcription factor, and this upregulates the invasion-promoting protein, HIC5. Indeed, we identified Sox4 as the most anti-correlated predicted target of miR-29a in glioblastoma. Importantly, inhibition of endogenous miR-29a decreases glioblastoma growth and invasion in vitro and in vivo, and increased miR-29a expression in glioblastoma specimens correlates with decreased patient survival.

**Conclusions:**

Taken together, these data identify miR-29a as a master regulator of glioblastoma growth and invasion.

**Electronic supplementary material:**

The online version of this article (10.1186/s13046-019-1026-1) contains supplementary material, which is available to authorized users.

## Background

MicroRNAs are short (about 22 nucleotides) non-coding RNAs that generally repress translation by targeting complementary sequences in the 3′ untranslated region (3’-UTR) of messenger RNAs [1]. A single microRNA can target dozens of messenger RNAs, thereby regulating complex biological processes. Numerous reports have detailed the important role that microRNAs play in development and in carcinogenesis [2–4]. Because the function of microRNAs is determined in part by the co-expression of their specific target mRNAs, their roles are complex and tissue specific. One striking example of this tissue-specific complexity is miR-29a, which has widely been reported to be a tumor suppressor in acute myeloid leukemia [5], lymphoma [6–8], hepatocellular carcinoma [9, 10] and gastric cancer [11, 12]. However, other studies have reported an oncogenic role for miR-29a in acute myeloid leukemia [13] and chronic lymphocytic leukemia [14]. Likewise, miR-29a has been reported to either decrease invasion in human carcinoma cell lines [15] or to increase invasion in human epithelial cancers [16] and in human hepatoma cells [17]. Thus, it is essential that the role of individual microRNAs such as miR-29a be evaluated in their native context, as tissue-specific or cell type-specific gene expression patterns have a tremendous impact on their function.

One of the cancers where microRNAs have been shown to play an important role is glioblastoma, the most common and most malignant intrinsic brain tumor [18–21]. Despite treatment, the median survival of patients with glioblastoma is only 14 to16 months and, at present, there is no cure [22]. Recent studies have used molecular features to divide glioblastomas into several subclasses [19, 23–25]. We recently used microRNA expression profiles to classify glioblastoma into five genetically and clinically distinct subclasses, and showed that microRNAs contribute significantly to the phenotypic characteristics of each subclass [19]. Although a growing number of microRNAs have been implicated in glioblastoma, the functional role of a majority of these molecules remains unknown.

Here we report that miR-29a is expressed primarily in the most aggressive glioblastoma subclasses, and its expression correlates with short patient survival. miR-29a downregulates PTEN, EphB3 and SOX4 expression to activate a complex post-transcriptional program of growth and invasion in glioblastoma. Antagonism of miR-29a inhibits glioblastoma growth and invasion in vitro and in vivo, suggesting that this approach may represent a novel therapeutic strategy in glioblastoma.

## Methods

### Lentiviruses and cell lines

All studies involving primary human tissues were conducted under the auspices of a human subjects protocol approved by the Institutional Review Board at Brigham and Women’s Hospital. Primary glioblastoma stem-like cells were prepared from surgical glioblastoma specimens as described previously [26]. Human U87, U251 and LN229 glioblastoma cell lines were purchased from the American Tissue Type Culture Collection. The hsa-miR-29a sequence with ~ 264 bp of flanking sequence was cloned from human genomic DNA by PCR and confirmed by DNA sequencing. The forward primer was 5′-gcacctcgattagttctcg-3′, and the reverse primer was 5′-ccaagctggcctaacttcag-3′. The PCR product was transferred into the pLenti6-IRES-GFP vector and packaged in 293FT cells [19]. A control EGFP lentivirus lacking a microRNA sequence was also prepared. miR-29a sponge (miR-Locker) and control sponge lentiviral vectors were purchased from Biosettia and packaged in 293FT cells. LN229 and U251 glioblastoma (GBM) cells were transduced with the control, miR-29a or miR-29a sponge lentiviruses, and stable cell lines were selected using blasticidin.

### Genome-scale expression data and analysis

Array comparative genomic hybridization (CGH), microRNA, mRNA and clinical data for 197 GBM patients were downloaded from The Cancer Genome Atlas project data portal (http://cancergenome.nih.gov) in March 2009. Data use certification was obtained for the use of controlled-access data. Details on the processing and platforms used, as well as the methods for selection of highly informative microRNAs and consensus clustering are as described previously [19]. In brief, we used the online website http://www.microrna.org/microrna/home.do to identify potential targets and looked at other anti-correlative targets using The Cancer Genome Atlas (TCGA) dataset. Additional data from TCGA for Kaplan-Meier survival analyses for Sox4 and HIC5 was obtained at http://hgserver1.amc.nl/cgi-bin/r2/main.cgi.

### Luciferase reporter assays

Reporter constructs were generated by overexpressing a vector in which firefly luciferase was fused to the Sox4 3’-UTR containing the putative miR-29a binding site (Addgene). Expression of the luciferase fusion protein in 293 T cells was then determined as we have described previously [27] in the presence of a miR-29a mimic (100 nM), a control oligonucleotide (100 nM) or a miR-335 mimic (as a positive control).

### RNAi studies

A miRIDIAN double-stranded RNA miR-29a mimic and a hairpin inhibitor (antagomiR) for miR-29a, as well as the corresponding negative controls, were purchased from Dharmacon. SOX4 siRNA and a matched oligonucleotide control were purchased from Invitrogen. The miR-29a mimic or inhibitor was added to the medium at a concentration of 100 nM without the use of additional transfection reagents for 48 h prior to performing assays. For SOX4, EphB3 and HIC5 siRNA experiments, the siRNAs were purchased from Ambion. Oligofectamine was used to transiently transfect the cells overnight prior to performing siRNA assays.

### Real-time polymerase chain reaction (PCR)

Total RNA enriched for microRNA was extracted from LN229 or U251 GBM cell lines using a commercially available kit (Qiagen). cDNA was then prepared using 1 μg of total RNA from each sample (SuperScript III First-Strand Synthesis SuperMix, Invitrogen). A miR-29a-specific Real-time PCR primer was purchased from Applied Biosystems. Six nanograms of cDNA were used for real-time PCR analysis in a final reaction volume of 20 uL. The samples were analyzed in triplicate using an ABI 7300 Real-time PCR machine, and statistical analysis was performed using the t test.

### Western blots

Western blots were performed as described previously [27]. Briefly, total protein was extracted by RIPA buffer and separated by gel electrophoresis. The protein was then transferred to nitrocellulose membranes and probed overnight using the appropriated primary antibodies. The antibodies used were PTEN, SOX4, EphB3 (Abcam); HIC5 (BD Biosciences); AKT, phosphorylated AKT (ser473), GSK3β, phosphorylated GSK3β (ser9), (Cell Signaling); β-catenin and β-actin (Sigma). After incubation in the appropriate secondary antibodies, immunoreactive bands were visualized using chemiluminescence.

### In vitro proliferation, growth, apoptosis and invasion assays

Cell growth or cell survival after DNA damage were assayed in vitro using the MTT assay, and cell proliferation was measured using bromodeoxy-uridine (BrdU) incorporation into DNA as we have described previously [27]. Matrigel transwell invasion assays were performed as we have described previously [28].

### In vivo tumor growth assay

All animal studies were conducted under the auspices of an IACUC protocol approved by the Harvard Medical Area Standing Committee on Animals. Human LN229 glioblastoma cells were transduced with either a miR-29a lentivirus or a control virus, and stable cell lines were established. 5 × 10^6^ control or miR-29a expressing glioblastoma cells were then injected subcutaneously into nude mice (*n* = 6 animals per condition). Subcutaneous tumor growth was then measured serially, and tumor volume was calculated using the formula for a spheroid. Significance was determined using an upaired t-test.

### Intracranial invasion assay

U251 glioblastoma cells were transduced with either a miR-29a sponge lentivirus encoding Green Fluorescent Protein (GFP) or a control sponge lentivirus encoding Red Fluorescent Protein (RFP). The green or red color was reinforced by DiO (green) or DiI (red) staining. Control and miR-29a sponge-expressing cells were mixed in a 1:1 ratio, and 2 × 10^5^ cells were then injected intracranially into nude mice. After 1 week, the animals were sacrificed and the brains were sectioned and processed for immunofluorescence imaging. The extent of radial invasion by glioblastoma cells transduced with the miR-29a sponge lentivirus (green) versus the control sponge lentivirus (red) was then assessed.

## Results

### miR-29a promotes glioblastoma growth

**Fig. 1:**
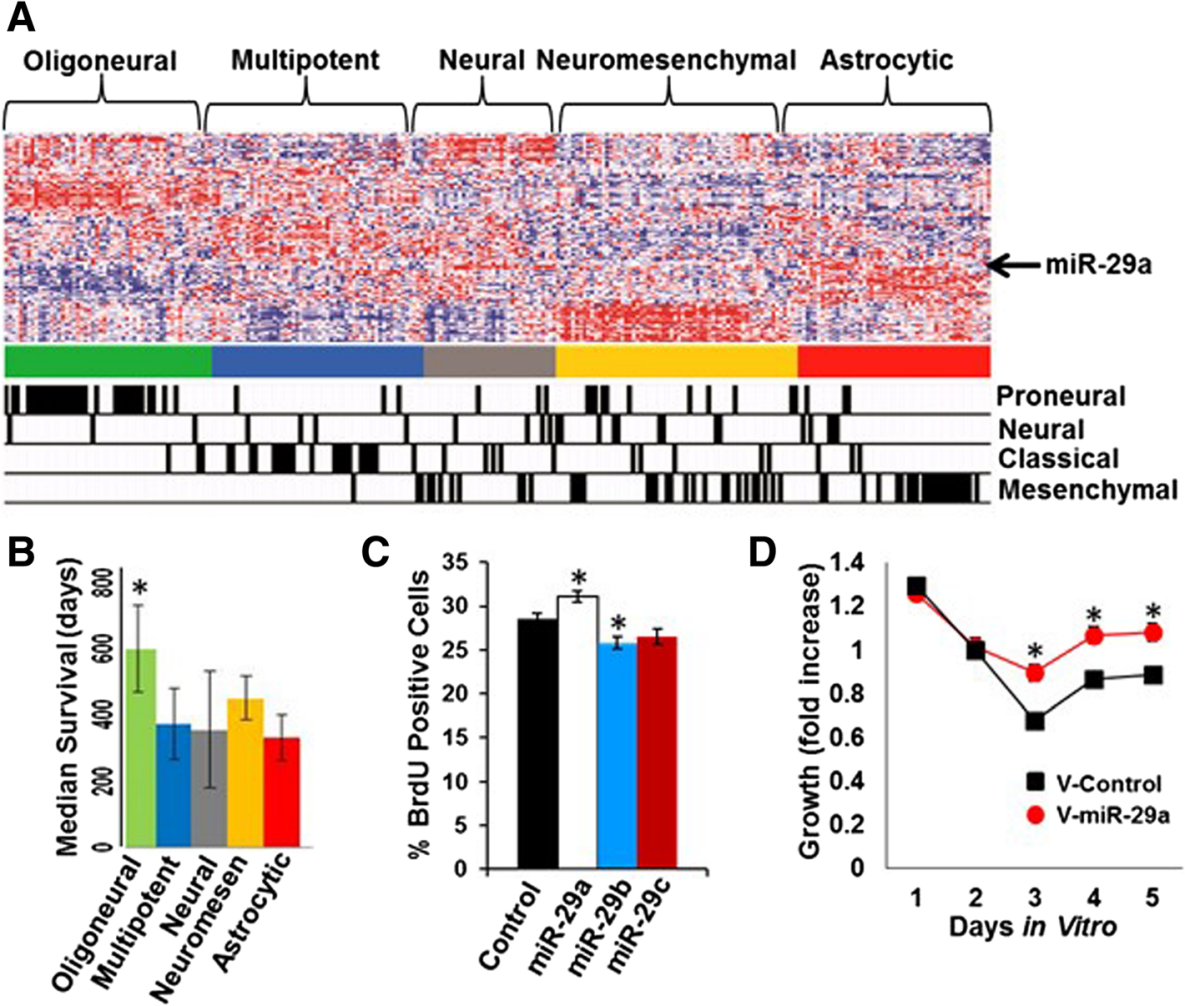
miR-29a is preferentially expressed in the most aggressive glioblastoma subclasses and promotes glioblastoma growth: **a**) Consensus clustering microRNA expression heatmap using 121 highly informative microRNA expression profiles from 197 glioblastomas demonstrating the expression of miR-29a (arrow) in the astrocytic and neural subclasses. The microRNA based classification of the tumor specimens is indicated at the top of the diagram, and the mRNA-based classification is indicated at the bottom. **b**) Median survival of each of the microRNA-based glioblastoma subclasses. **c**) BrdU proliferation assay illustrating the effect of miR-29a, miR-29b, miR-29c or a control mimic (100 nM) on proliferation in human U87 glioblastoma cells. Mean ± SEM (*P < 0.026, unpaired t-test). **d**) MTT cell growth assay illustrating the effect of lentiviral-mediated overexpression of miR-29a or a control sequence on the growth of primary human glioblastoma stem-like cells (*P < 0.0001, unpaired t-test)

We previously reported that patients with glioblastomas from the astrocytic and neural glioblastoma subclasses have the shortest survival among glioblastoma patients [19]. In the current study, we set out to identify microRNAs that contribute most significantly to the aggressive characteristics of these glioblastoma subclasses. We analysed microRNA expression profiles from 197 glioblastomas, and observed that members of the miR-29 subfamily (miR-29a, miR-29b, and miR-29c) were unique among the 171 informative microRNAs examined in that they showed a selective increase in expression in both the astrocytic and neural glioblastoma subclasses (Fig. 1A). Patients harboring glioblastomas from either of these two subclasses displayed the shortest median survival (Fig. 1B). We therefore examined the effects of microRNA mimics for miR-29a, miR-29b and miR-29c on proliferation in human U87 glioblastoma cells, and found that only the miR-29a mimic significantly increased proliferation (Fig. 1C, *P* = 0.026, unpaired t-test). Consequently, we selected miR-29a for further study.

**Fig. 2:**
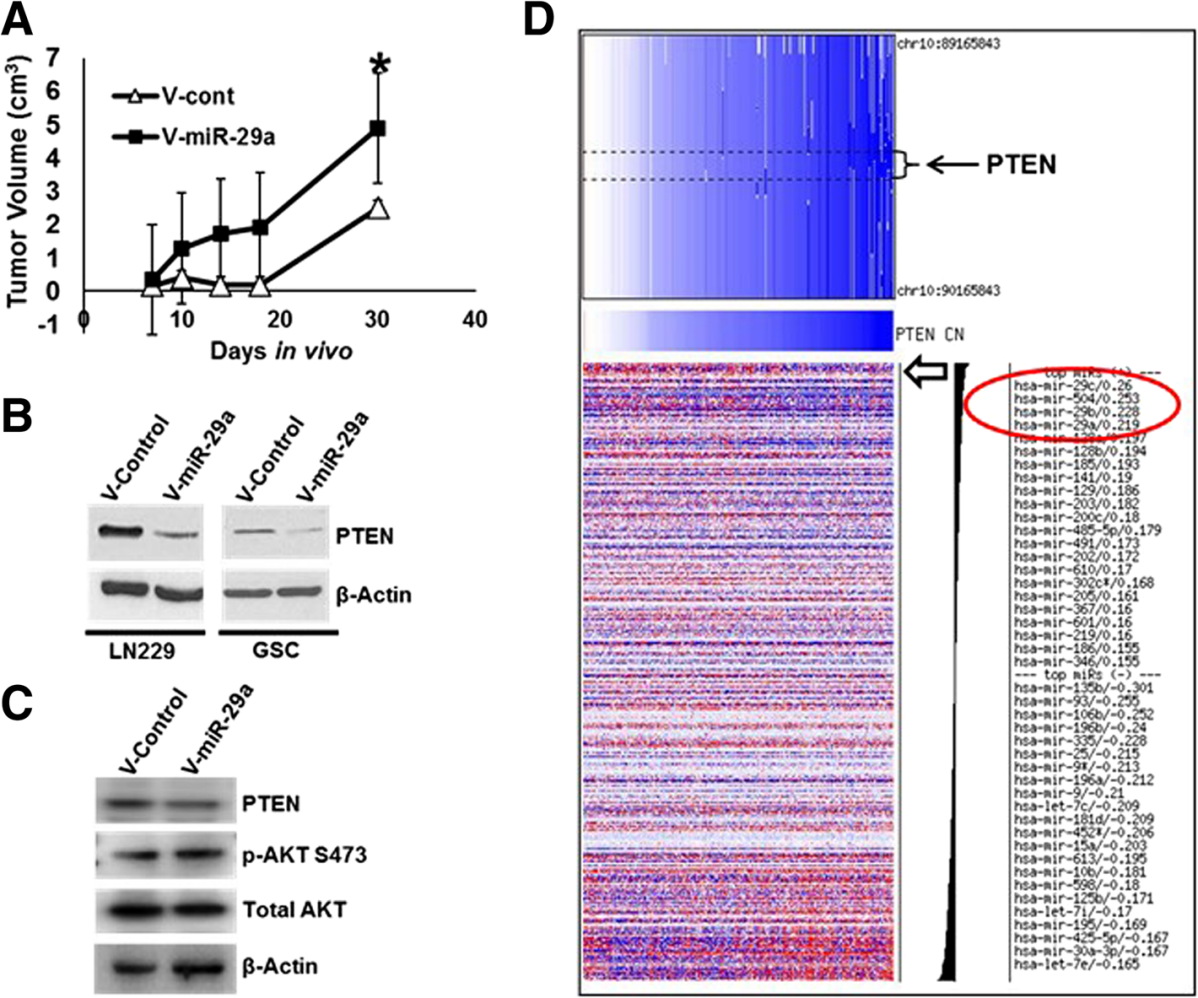
miR-29a downregulates PTEN in glioblastoma cells. **a**) LN229 glioblastoma cells transduced with a miR-29a lentivirus or a control virus were transplanted subcutaneously into nude mice (*n* = 8 animals/group), and tumor volume was measured serially. A significant increase in tumor growth was observed in LN229 glioblastoma cells overexpressing miR-29a (Mean ± SEM, *P < 0.05, unpaired t-test). **b**) Effect of miR-29a on PTEN expression in LN229 glioblastoma cells or in primary glioblastoma stem-like cells transduced with a control or miR-29a lentivirus. **c**) Western blot illustrating effect of miR-29a on PTEN expression and AKT phosphorylation (ser473) in LN229 glioblastoma cells transduced with a control or miR-29a lentivirus. **d**) Analysis of PTEN copy number and microRNA expression data for 470 microRNAs. Data obtained from the TCGA portal for 197 glioblastoma specimens. Circled area identifies miR-29a (PCC = 0.219), miR-29c (PCC = 0.260) and miR-29b (PCC = 0.228) as 3 of the top 4 microRNAs most positively correlated with PTEN copy number in glioblastoma. Open arrow indicates location of miR-29a expression profile

To determine the effect of miR-29a on glioblastoma tumor growth in vivo, we used lentiviral transduction to overexpress the miR-29a transcript in human LN229 glioblastoma cells. This afforded an approximately 2 fold increase in miR-29a expression in the cells (Additional file 1: Figure S1). Overexpression of miR-29a significantly increased the growth of LN229 glioblastoma cells in vitro (Fig. 1D, *P* < 0.02, unpaired t-test). LN229 glioblastoma cells transduced with the miR-29a lentivirus or a control lentivirus were subsequently transplanted subcutaneously into nude mice and tumor growth was monitored over time. LN229 glioblastoma cells overexpressing miR-29a formed significantly larger tumors than cells transduced with a control lentivirus (Fig. 2A, *P* < 0.05, unpaired t-test). These data indicated that miR-29a promotes glioblastoma growth.

### miR-29a targets PTEN in glioblastoma

We next investigated the mechanisms by which miR-29a increases glioblastoma growth. miR-29a has previously been reported to directly target the 3’-UTR of the PTEN tumor suppressor in hepatoma cells in vitro [17] and in neural stem cells [29]. PTEN is frequently mutated or deleted in glioblastoma, and we and others have reported that it is a target of oncogenic microRNAs in this tumor [30–32]. PTEN loss increases glioblastoma growth and invasion in part by activating the PI3 kinase/AKT pathway [33]. Western blot analysis indicated that miR-29a downregulates PTEN protein expression in LN229 glioblastoma cells and in primary glioblastoma stem-like cells (GSCs, Fig. 2B). As expected, the miR-29a-mediated repression of PTEN expression was accompanied by activation of AKT (Fig. 2C).

Integrated copy number, mRNA and microRNA expression analysis using data from 197 TCGA glioblastoma specimens failed to demonstrate an anti-correlation between mir-29a and PTEN mRNA expression (Pearson correlation coefficient (PCC) =0.038). Strikingly, however, miR-29a and its subfamily members (miR-29b and miR-29c) were foremost among microRNAs that were positively correlated with PTEN copy number (Fig. 2D, PCC = 0.219 for miR-29a). Thus, miR-29a is well positioned to suppress PTEN expression in glioblastomas in which the PTEN gene is intact.

### miR-29a decreases EphB3 to increase AKT in PTEN-deficient glioblastoma cells

**Fig. 3:**
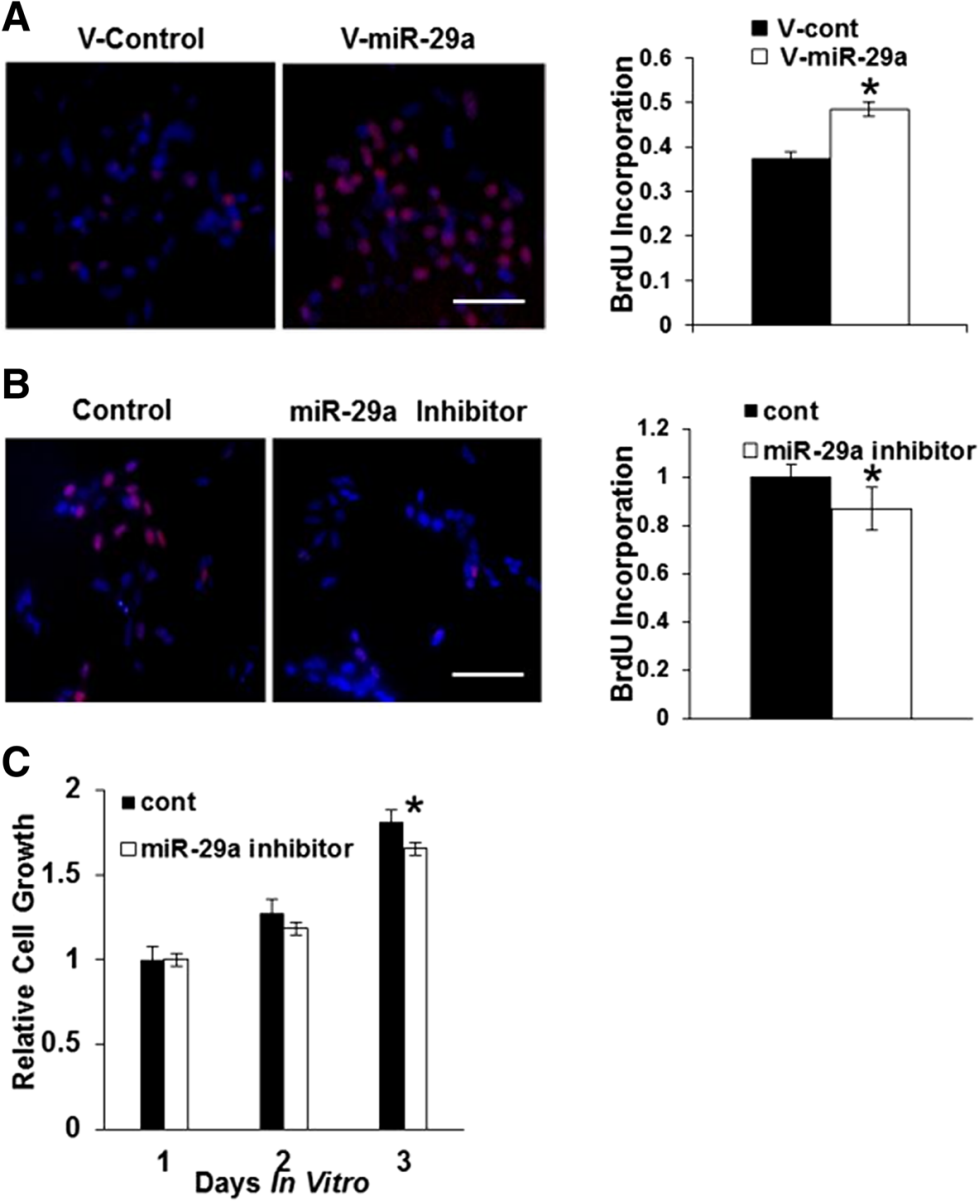
miR-29a increases proliferation and growth in glioblastoma cells lacking PTEN. **a**) Fluorescence micrographs (left panels) and data quantitation (right panel) from BrdU proliferation assay investigating the effect of lentiviral-mediated miR-29a overexpression on BrdU (red) incorporation into nuclei (DAPI, blue) by human U251 glioblastoma cells that lack functional PTEN. Data shown in right panel are mean ± SEM. *P < 0.0005, unpaired t-test. Scale approximately 100 μm. **b**) Fluorescence micrographs (left panels) and quantitation (right panel) from BrdU proliferation assay illustrating the effect of a miR-29a inhibitor (100 nM) on proliferation in PTEN-deficient U251 glioblastoma cells. *P* < 0.05, unpaired t-test. Data shown are mean ± SEM. *P < 0.0005, unpaired t-test. Scale approximately 100 μm. **c**) MTT cell growth assay illustrating the effect of a specific miR-29a inhibitor (100 nM) on the growth of PTEN-deficient U251 glioblastoma cells. Data shown are mean ± SEM. *P < 0.01, unpaired t-test

Our earlier finding that miR-29a increased the proliferation of human U87 glioblastoma cells (which lack functional PTEN) suggested the existence of additional mediators of miR-29a-induced glioblastoma growth (see Fig. 1C). To investigate this possibility further, we examined the effect of miR-29a on the growth of human U251 glioblastoma cells, which also lack functional PTEN. Lentiviral-mediated overexpression of miR-29a increased U251 glioblastoma cell proliferation significantly (Fig. 3A, *P* < 0.0005, unpaired t-test). Conversely, exposure of PTEN-deficient U251 cells to the miR-29a inhibitor (100 nM) significantly decreased proliferation (Fig. 3B, *P* < 0.05, unpaired t-test). Additionally, inhibition of endogenous miR-29a using the miR-29a inhibitor (100 nM) significantly decreased the growth of PTEN-deficient U251 glioblastoma cells (Fig. 3C, *P* < 0.01, unpaired t-test).

**Fig. 4:**
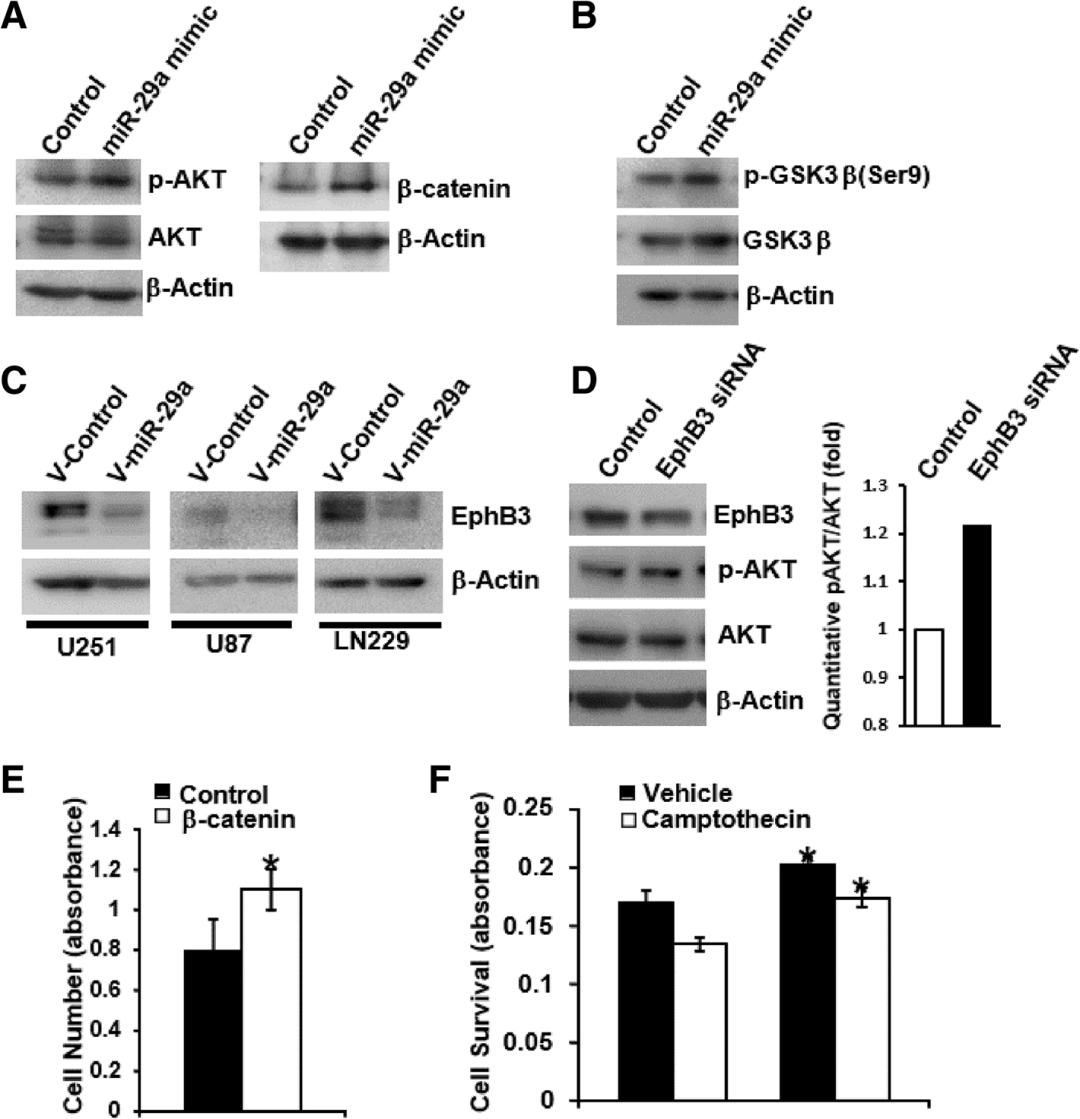
miR-29a activates AKT and increases β-catenin in PTEN-deficient glioblastoma cells. **a**) Effect of miR-29a mimic (100 nM) or a scrambled control mimic on AKT and β-catenin expression in PTEN-deficient U251 glioblastoma cells. **b**) Effect of miR-29a mimic (100 nM) or a control mimic on GSK3β phosphorylation in PTEN-deficient U251 glioblastoma cells. **c**) Effect of miR-29a on EphB3 expression in U251, U87 or LN229 glioblastoma cells transduced with a control or a miR-29a lentivirus. **d**) Western blot illustrating effect of EphB3 siRNA on EphB3 expression and AKT phosphorylation in LN229 glioblastoma cells. **e**) MTT assay illustrating effect of β-catenin overexpression on cell growth in human glioblastoma cells. Data shown are mean ± SEM. *P < 0.05, unpaired t-test. **f**) MTT assay illustrating effect of the miR-29a mimic (100 nM) on survival of U251 glioblastoma cells after camptothecin exposure (75 nM; mean ± SEM, *P < 0.006, unpaired t-test)

In order to identify growth-promoting pathways activated by miR-29a in the absence of PTEN, we exposed PTEN-deficient U251 glioblastoma cells to a miR-29a mimic (100 nM), collected the protein and examined the lysates using an antibody array that assays several key growth regulatory pathways in the cell (Human Phospho-Kinase Array Kit, R&D Systems). This assay revealed that miR-29a increased AKT phosphorylation and β-catenin expression in U251 glioblastoma cells, and this was confirmed by Western blot (Fig. 4A). AKT can phosphorylate and inactivate GSK3β [34] which, in turn, phosphorylates β-catenin and targets it for degradation [35]. Indeed, miR-29a induced inhibitory phosphorylation of GSK3β on serine 9 (Fig. 4B), suggesting that the increased β-catenin expression observed in the presence of miR-29a may result from AKT-dependent phosphorylation and inhibition of GSK3β activity.

In order to identify upstream mediators of the effect of miR-29a on AKT activation, we identified predicted anti-correlated mRNA targets of miR-29a using microRNA and mRNA expression profiles from 261 TCGA glioblastoma specimens. We identified EphB3 as an anti-correlated (PCC = − 0.508) predicted target of miR-29a. EphB3 encodes a receptor tyrosine kinase that suppresses AKT activation in lung cancer cells [26]. Western blot confirmed decreased EphB3 expression in human U251, LN229 and U87 cells transduced with the miR-29a lentivirus (Fig. 4C). Moreover, siRNA-mediated knockdown of EphB3 in LN229 glioblastoma cells increased AKT phosphorylation and activation (Fig. 4D). Thus, miR-29a decreases EphB3 expression to activate AKT in glioblastoma.

In addition to their effects on proliferation, both AKT and β-catenin can inhibit apoptosis in glioblastoma cells [26, 27]. Enforced overexpression of β-catenin increased glioblastoma cell growth (Fig. 4E, *P* < 0.05, unpaired t-test). We therefore examined the effect of miR-29a on the survival of PTEN-deficient U251 glioblastoma cells after DNA damage. The miR-29a mimic (100 nM) significantly increased cell growth under basal conditions and increased survival after exposure to the DNA-damaging agent, camptothecin (Fig. 4F, *P* < 0.006, unpaired t-test). Taken together, these data suggest that miR-29a decreases EphB3 to activate the PI3K/AKT and Wnt pathways, thereby promoting proliferation and survival in glioblastoma cells.

### miR-29a targets Sox4 to promote invasion in PTEN-deficient glioblastoma cells

**Fig. 5:**
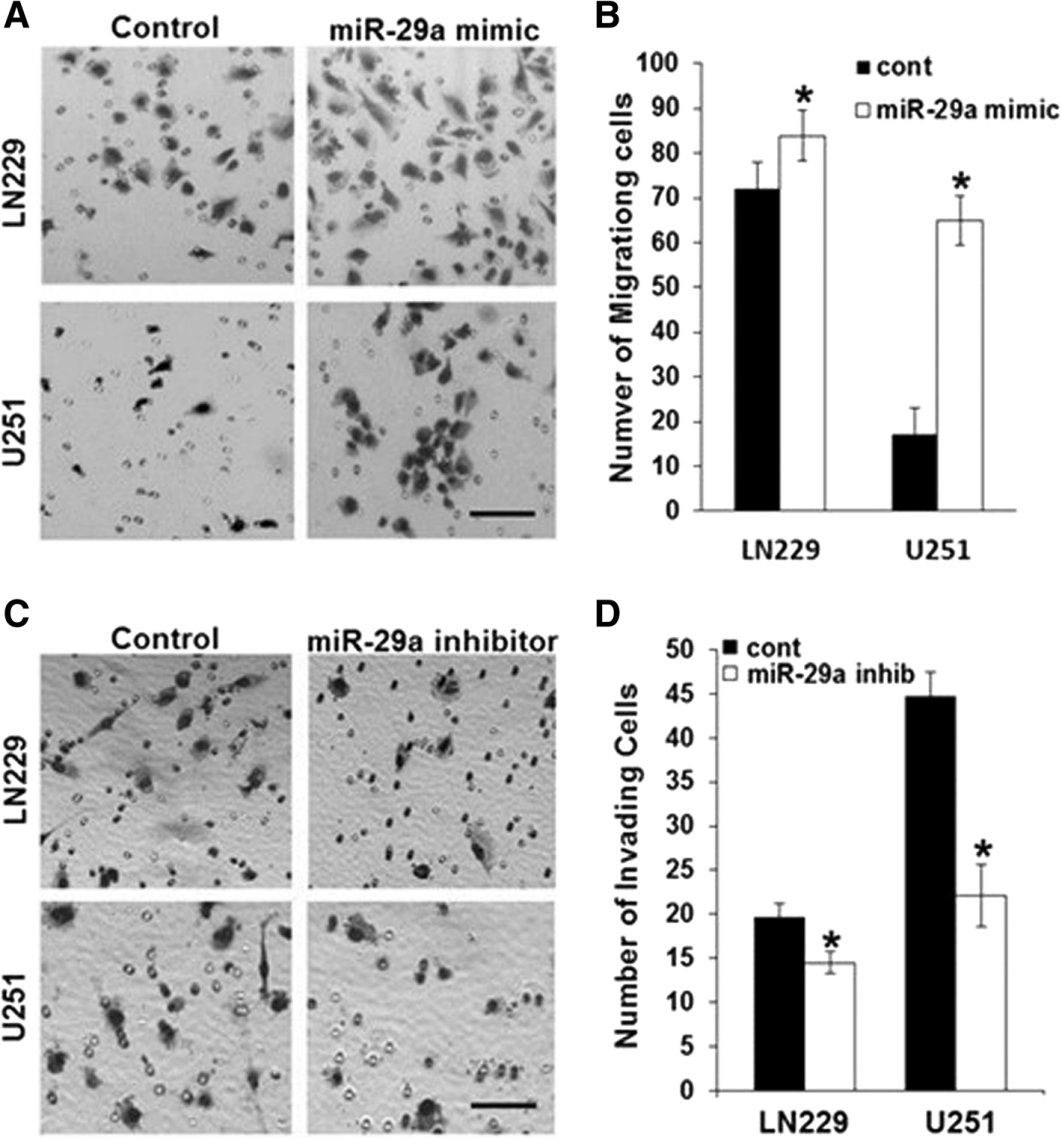
miR-29a robustly increases invasion in PTEN-competent and PTEN-deficient glioblastoma cells. **a**) Phase micrographs of invading LN229 or U251 glioblastoma cells in Matrigel invasion assay after exposure to control or miR-29a mimic (100 nM). Scale approximately 50 μm. **b**) Quantitation of data depicted in **a**). Data shown are mean ± SEM of 6 replicates. *P < 0.05 for LN229 and *P < 0.0001 for U251 glioblastoma cells. c) Phase micrographs of invading LN229 or U251 glioblastoma cells in Matrigel invasion assay after exposure to control or miR-29a inhibitor (100 nM). Scale approximately 50 μm. **d**) Quantitation of data depicted in **c**). Data shown are mean ± SEM of 6 replicates. *P < 0.01 for LN229 and *P < 0.0001 for U251 glioblastoma cells

PTEN is a major regulator of invasion in glioblastoma and other cancers [36]. Our finding that miR-29a targets PTEN in glioblastoma suggests that miR-29a also promotes glioblastoma invasion. Indeed, miR-29a has recently been reported to enhance hepatoma cell migration by targeting PTEN [17]. However, many glioblastomas lack functional PTEN, raising the question of whether miR-29a differentially regulates invasion in PTEN-competent and PTEN-deficient glioblastomas. Exposure of human LN229 (PTEN-competent) or U251 (PTEN-deficient) glioblastoma cells to a miR-29a mimic (100 nM) significantly increased glioblastoma cell invasion by both cell lines in a 3-D Matrigel invasion assay (Fig. 5A, B, *P* < 0.0001, unpaired t-test). In addition, miR-29a increased invasion in primary glioblastoma stem-like cells (Additional file 1: Figure S2). A miR-29a antagomiR (100 nM) decreased invasion in both LN229 (*P* < 0.015, unpaired t-test) and U251 (*P* < 0.0001, unpaired t-test) glioblastoma cells (Fig. 5C, D). Thus, miR-29a promotes glioblastoma cell invasion in PTEN-competent and PTEN-deficient glioblastomas.

Like PTEN, EphB3 suppresses AKT activation and inhibits lung cancer cell migration [37]. Although AKT activation can promote glioblastoma cell invasion, β-catenin reportedly inhibits invasion in these cells [38]. Our finding that miR-29a decreases expression of both PTEN and EphB3 in glioblastoma raised the possibility that miR-29a regulates a coordinated invasion program in glioblastoma. We therefore searched for additional miR-29a targets that might mediate its effects on glioblastoma invasion. Computational analysis of microRNA and mRNA expression profiles from 261 primary glioblastoma specimens identified Sox4 as the most anti-correlated predicted mRNA target of miR-29a (PCC = − 0.636). Likewise, miR-29a was the microRNA that was most anti-correlated with Sox4 (Additional file 1: Figure S3). Sox4 is an HMG box transcription factor that regulates a variety of biological processes, including neural differentiation [39]. Numerous but conflicting reports indicate that Sox4 may act as either an oncogene or a tumor suppressor in a range of cancers [39]. Relevant to the current study is a previous report that loss of Sox4 promotes melanoma cell invasion via a mechanism that involves activation of NFκB [40, 41].

**Fig. 6:**
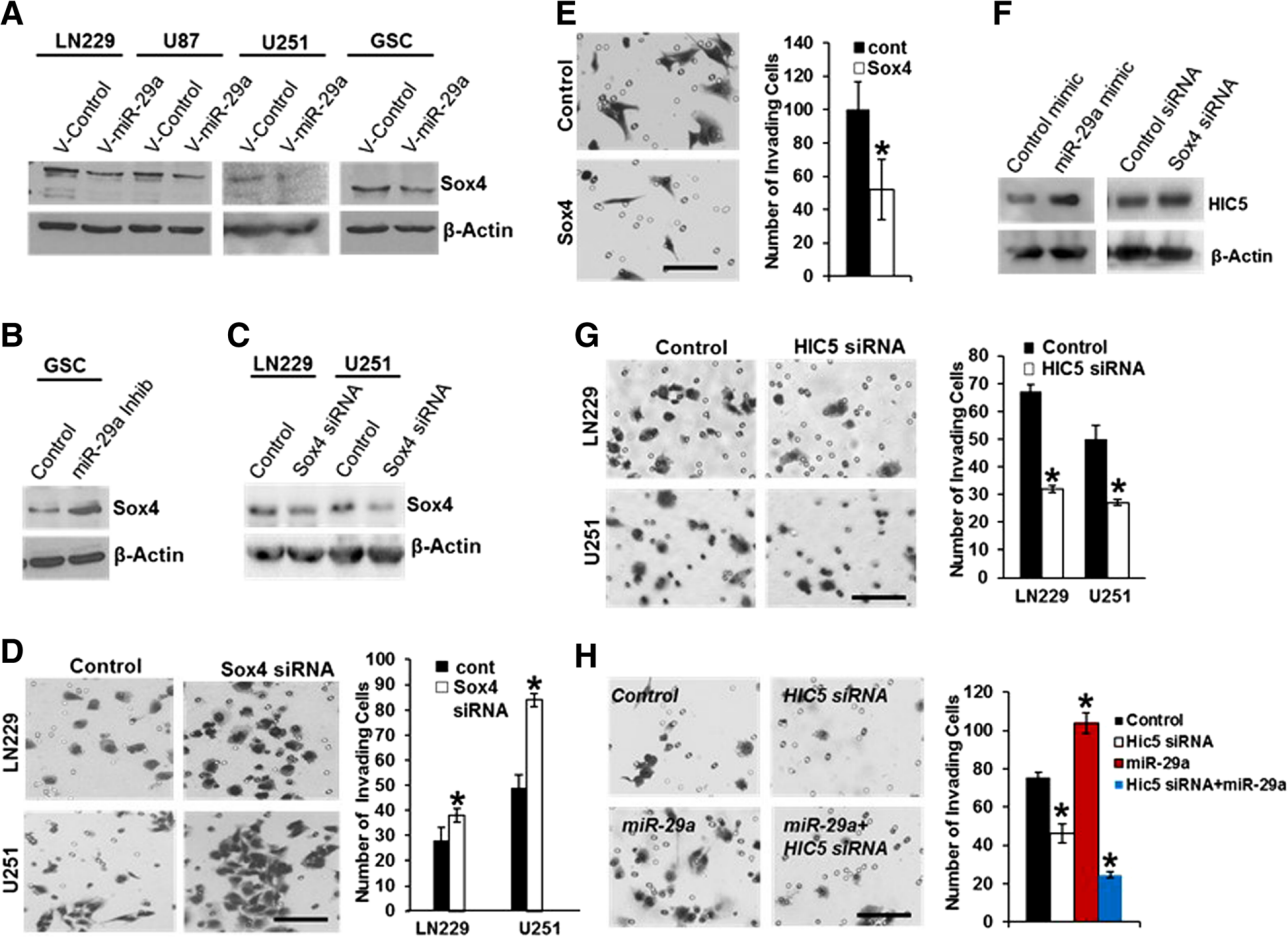
miR-29a activates a Sox4/HIC5 invasion pathway in glioblastoma. **a**) Effect of lentiviral-mediated overexpression of miR-29a on Sox4 expression in LN229, U87, U251 or primary human glioblastoma stem-like cells (GSC). **b**) Effect of miR-29a antagomiR (100 nM) on Sox4 protein expression in primary human glioblastoma stem-like cells. **c**) Western blot illustrating Sox4 knockdown using siRNA transfection in LN229 or U251 glioblastoma cells. **d**) Phase micrographs (left panels) or quantitation of data (right panel) from a Matrigel invasion assay showing effect of siRNA-mediated Sox4 knockdown on LN229 or U251 glioblastoma cell invasion. Mean ± SEM. *P* < 0.0001, unpaired t-test. Scale approximately 50 μm. **e**) Phase micrographs (left panels) or data quantitation (right panel) from a 3D Matrigel invasion assay showing effect of Sox4 overexpression on PTEN-deficient U87 glioblastoma cell invasion. Mean ± SEM; *P* < 0.01, unpaired t-test. Scale approximately 50 μm. **f**) Effect of miR-29a mimic or Sox4 siRNA on HIC5 protein expression in human U251 glioblastoma cells. **g**) Phase micrographs (left panels) or data quantitation (right panel) from a Matrigel invasion assay showing effect of siRNA-mediated HIC5 knockdown on LN229 or U251 glioblastoma cell invasion. Mean ± SEM. *P < 0.0001, unpaired t-test. Scale approximately 50 μm. **h**) Phase micrographs (left panels) or data quantitation (right panel) from a Matrigel invasion assay showing effect of siRNA-mediated HIC5 knockdown on invasion induced by miR-29a in human U251 glioblastoma cells. Mean ± SEM. *P < 0.0001, unpaired t-test. Scale approximately 50 μm

Western blot analysis indicated that miR-29a robustly downregulates Sox4 protein expression in multiple human glioblastoma cell lines and in primary glioblastoma stem-like cells (Fig. 6A), and the miR-29a inhibitor (100 nM) increased Sox4 protein levels in primary glioblastoma stem-like cells (Fig. 6B). Real-time PCR revealed a miR-29a-induced decrease in Sox4 mRNA levels (Additional file 1: Figure S1). Importantly, a luciferase reporter assay in which the SOX4 3’-UTR was fused to the firefly luciferase mRNA sequence indicated that miR-29a directly targets the Sox4 3’UTR (Additional file 1: Figure S3).

We depleted endogenous Sox4 protein expression via transient transfection of Sox4 siRNA in LN229 or U251 glioblastoma cells (Fig. 6C), and found that this significantly increased glioblastoma cell invasion (Fig. 6D, *P* < 0.0001, unpaired t-test). Conversely, Sox4 overexpression in PTEN-deficient U87 glioblastoma cells decreased invasion (Fig. 6E, *P* < 0.01, unpaired t-test). Manipulation of Sox4 expression did not increase AKT phosphorylation or β-catenin levels (data not shown). Taken together, these data indicate that miR-29a targets Sox4 to promote glioblastoma cell invasion independent of PTEN.

### HIC5 is a downstream mediator of the mir-29a/Sox4 invasion pathway in glioblastoma

Loss of Sox4 has been reported to promote invasion by activating the NFκB pathway [40, 42]. However, the miR-29a mimic failed to promote NFκB nuclear translocation in human glioblastoma cells, suggesting that NFκB activation is not responsible for the miR-29a-induced increase in glioblastoma cell invasion (data not shown). To identify other possible downstream mediators of the miR-29a/Sox4 invasion pathway, we queried public databases [43, 44] to search for Sox4 regulated transcripts that have been implicated in cell migration or invasion. We identified HIC5 (a.k.a. TGFβ1I1) as a migration-related transcript [45–48] that is upregulated after knockdown of Sox4 in other cell types. Western blot analysis revealed upregulation of HIC5 protein expression after exposure of human U251 glioblastoma cells to the miR-29a mimic or after siRNA-mediated knockdown of Sox4 (Fig. 6F). Knockdown of HIC5 expression using siRNA significantly inhibited LN229 (PTEN-competent) and U251 (PTEN-deficient) glioblastoma cell invasion (Fig. 6G, *P < 0.0001, unpaired t-test). Moreover, knockdown of HIC5 completely abrogated the increase in invasion induced by miR-29a in PTEN-deficient U251 cells, suggesting that it plays an essential role in this process (Fig. 6H).

### miR-29a regulates glioblastoma invasion in vivo and correlates with survival

Our earlier studies using human LN229 glioblastoma cells transplanted subcutaneously unto nude mice indicated that miR-29a promotes glioblastoma tumor growth (see Fig. 2A). We next investigated the effect of endogenous miR-29a on glioblastoma invasion in vivo using an intracranial human glioblastoma xenograft mouse model. PTEN-deficient human U251 glioblastoma cells were transduced with a lentivirus containing a nucleotide sequence complementary to miR-29a (miR-29a sponge/miR-locker) or a control sequence (control sponge/miR-locker), and a stable cell line was then selected. The control and miR-29a sponge lentiviral vectors also encoded either Red Fluorescent Protein (control) or Green Fluorescent Protein (miR-29a), respectively. Overexpression of the bulged miR-29a sponge sequence increased mir-29a levels (Additional file 1: Figure S1), presumably because it interfered with RISC-mediated degradation of the miRNA/mRNA target duplex. The sponge effectively antagonized the ability of miR-29a to degrade its mRNA targets, as evidenced by the elevation of Sox4 mRNA (Additional file 1: Figure S1). This elevation was in contrast to the decrease in Sox4 mRNA expression induced by miR-29a itself (Additional file 1: Figure S1).

**Fig. 7:**
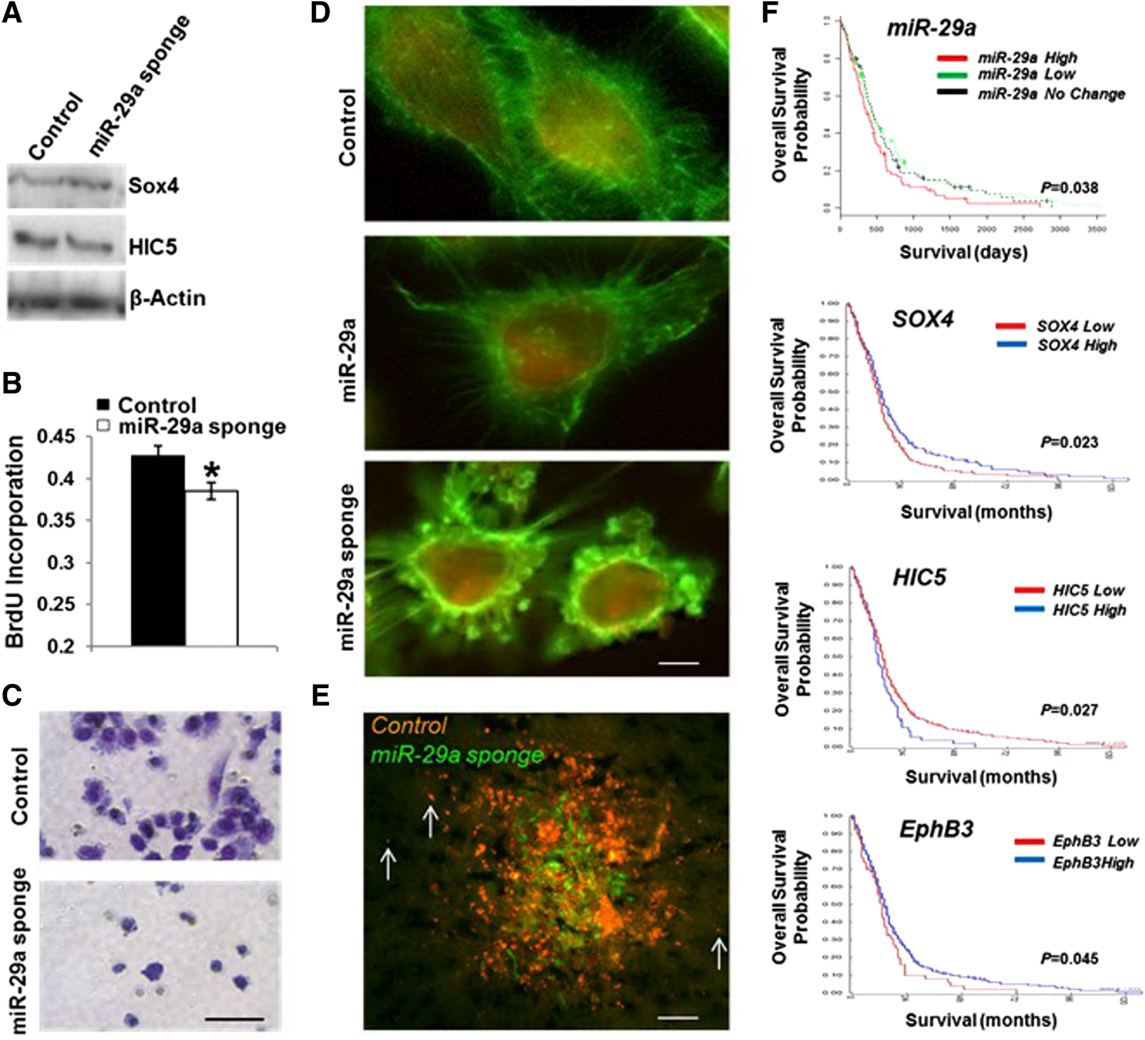
Endogenous miR-29a regulates glioblastoma cell invasion in vivo and correlates with patient survival. **a**) Effect of miR-29a sponge overexpression on Sox4 and HIC5 expression in human U251 glioblastoma cells. **b**) BrdU proliferation assay illustrating the effect of a miR-29a sponge on U251 glioblastoma cell proliferation. Mean ± SEM. *P < 0.02, unpaired t-test. **c**) Matrigel invasion assay illustrating effect of miR-29a sponge on U251 glioblastoma cell invasion. **d**) Fluorescence micrographs of U251 glioblastoma cells transduced with lentiviruses encoding miR-29a, a miR-29a sponge or a control sequence. Scale approximately 3 μm. **e**) Fluorescence micrograph of mouse brain section obtained 1 week after transplantation of U251 glioblastoma cells transduced with a control (red) or miR-29a sponge lentivirus. Scale approximately 500 μm. **f**) Kaplan-Meier survival analyses using microRNA expression profiles (*n* = 197) or mRNA expression profiles (*n* = 504) obtained from the TCGA portal for glioblastoma. *P* values are miR-29a (*P* = 0.038), SOX4 *(P* = 0.023), HIC5 (*P* = 0.027), EphB3 (*P* = 0.045)

Overexpression of the miR-29a sponge increased Sox4 protein expression and decreased HIC5 protein expression in U251 glioblastoma cells (Fig. 7A). In addition, it significantly decreased U251 glioblastoma cell proliferation (Fig. 7B, *P* < 0.02, unpaired t-test. The miR-29a sponge also inhibited cell growth (*P* < 0.0001, unpaired t-test) and increased DNA damage-induced apoptosis (*P* < 0.05, unpaired t-test) in U251 glioblastoma cells in vitro (Additional file 1: Figure S1).

Inhibition of endogenous miR-29a using the miR-29a sponge significantly decreased glioblastoma cell invasion in vitro (Fig. 7C and Additional file 1: Figure S5). We examined the effect of miR-29a on glioblastoma cell morphology using human U251 glioblastoma cells transduced with control, miR-29a or miR-29a lentiviruses. When compared to control U251 glioblastoma cells, cells overexpressing miR-29a were smaller and displayed moderately fewer filopodia (Fig. 7D). In contrast, cells overexpressing the miR-29a sponge adopted a rounded morphology with a marked reduction in filopodia and lamellopodia (Fig. 7D).

In order to investigate the role of miR-29a in glioblastoma cell invasion in vivo, PTEN-deficient U251 glioblastoma cells expressing either the control (RFP) or mir-29a (GFP) sponges were mixed 1:1 and injected intracranially into the brains of nude mice. After one week, the brains were collected and processed for fluorescence imaging to identify invading cells. Glioblastoma cells overexpressing the miR-29a sponge (green fluorescence) migrated from the injection site less than control cells (red fluorescence, Fig. 7E).

Our initial observations using primary glioblastoma specimens indicated that miR-29a is preferentially expressed in the astrocytic and neural glioblastoma subclasses. Because these subclasses display the shortest median survival among the five glioblastoma subclasses identified by microRNA profiling, our findings suggested that miR-29a may be associated with decreased patient survival. Indeed, Kaplan-Meier survival analysis using microRNA expression data from 261 primary glioblastoma specimens obtained from the TCGA portal indicated that increased miR-29a expression is associated with decreased patient survival (Fig. 7F, *P* = 0.038, Logrank). Consistent with the miR-29a/Sox4/HIC5 invasion pathway identified by our in vitro studies, increased Sox4 mRNA expression is positively correlated with patient survival (Fig. 7F, *P* = 0.023, Logrank), and HIC5 mRNA expression is negatively correlated with survival (Fig. 7F, *P* = 0.027, Logrank). Of note, decreased EphB3 mRNA expression also correlated with decreased survival (Fig. 7F, *P* = 0.045, Logrank). Taken together, these data establish a role for endogenous miR-29a in glioblastoma growth and invasion.

## Discussion

MicroRNA-29a is a conserved microRNA that is involved in the regulation of several coordinated post-transcriptional programs affecting different biological processes. For example, miR-29a represses the translation of multiple extracellular matrix proteins, and miR-29a depletion leads to fibrosis in several tissues [49]. miR-29a also regulates the myeloid differentiation program [5]. We report here that miR-29a regulates a complex program of cell growth and invasion in glioblastoma. This program not only involves co-activation of the AKT/PI3K and Wnt pathways via downregulation of PTEN and EphB3, but also activation of a newly discovered Sox4/Hic5 invasion pathway (Additional file 1: Figure S6).

MicroRNA-29a has previously been reported to promote hepatoma cell migration by directly targeting PTEN, a key regulator of migration in many cell types [17, 50]. We observed that miR-29a robustly downregulates PTEN in glioblastoma cells that have intact PTEN function. Surprisingly, however, we did not find an anti-correlation between miR-29a and PTEN mRNA expression. This may be due in part to the impact of other mechanisms that regulate PTEN expression and function in glioblastoma, including deletions, mutations and the impact of other microRNAs [19, 27, 31, 32, 51]. Interestingly, miR-29a is among the top 1% of microRNAs in terms of its positive correlation with PTEN copy number in glioblastoma, suggesting that miR-29a-mediated downregulation of PTEN provides a selective growth advantage in glioblastoma cells with intact PTEN.

In many glioblastomas, PTEN is deleted or mutated [51]. In such tumors, we find that miR-29a nevertheless promotes growth by downregulating EphB3, thereby increasing AKT activation and β-catenin levels. Activation of the EphB3 receptor leads to PP2A-mediated dephosphorylation and inactivation of AKT [37]. Interestingly, EphB3 also inhibits the migration of lung cancer cells [37].

Consistent with previous reports [34], we find that AKT activation is accompanied by phosphorylation and inactivation of GSK3β which, in turn, phosphorylates β-catenin and targets it for degradation [35]. In osteoblasts, miR-29a has also been reported to increase β-catenin by directly targeting several Wnt pathway antagonists other than GSK3β [52]. Additional studies are needed to determine whether miR-29a also increases β-catenin via these mechanisms in glioblastoma.

In addition to the AKT/PI3 kinase and Wnt pathways, miR-29a activates a newly-discovered Sox4/Hic5 invasion pathway in glioblastoma cells. This pathway operates in PTEN-deficient glioblastoma cells to robustly promote invasion. Sox4 is an HMG transcription factor that promotes neuronal differentiation during nervous system development [53]. Recent reports indicate that decreased Sox4 expression promotes invasion in melanoma [39, 40]. This effect is thought to involve activation of the NFκB pathway and regulation of DICER expression. In the current study, we did not find evidence for miR-29a-induced nuclear translocation of NFκB, suggesting that this is not the mechanism underlying miR-29a-induced invasion in glioblastoma. However, we did observe robust upregulation of HIC5 after miR-29a exposure or Sox4 knockdown in glioblastoma cells. Increased HIC5 expression promoted glioblastoma cell invasion, and HIC5 knockdown abrogated the miR-29a-induced increase in invasion. HIC5 is homologous to paxillin and associates with the focal adhesion kinases, FAK and Pyk2, at focal contacts [45, 46]. HIC5 has also been reported to promote epithelial-to-mesenchymal transition (EMT) and invadopodia formation by regulating the Rho/ROCK pathway [11, 47].

However, as a tumor promoter, miR-29 mediates epithelial-mesenchymal transition (EMT) and promotes metastasis in breast cancer, colon cancer and pancreatic cancer [54–56]. There is also evidence that miR-29 may regulate MMP2 or Mcl-1 [56, 57], which partly participate in the process of metastasis, yet the underlying mechanisms remain controversial. Interestingly, we saw no evidence of Rac1 activation by miR-29a (personal observations). Additional studies are thus underway to determine the downstream signaling pathway activated by HIC5 in glioblastoma cells.

## Conclusions

Our findings have potentially significant clinical implications. miR-29a expression is high in the astrocytic glioblastoma subclass, and this distinguishes this subclass from the other mesenchymal glioblastoma subclass (i.e. the neuromesenchymal subclass). Thus, miR-29a may contribute to the decreased survival of patients with glioblastomas from the astrocytic subclass. Sox4 is the most anti-correlated predicted target of miR-29a in glioblastoma and is positively correlated with survival, while HIC5 expression is upregulated by miR-29a or by decreased Sox4, and is anti-correlated with survival. This pattern suggests that the miR-29a/Sox4/HIC5 invasion pathway is functional in glioblastomas in vivo. Our finding that miR-29a increases growth and invasion by downregulating PTEN, EphB3 and Sox4 in glioblastoma indicates the presence of a coordinated program promoting tumor aggressiveness. Importantly, inhibition of endogenous miR-29a decreases glioblastoma growth and invasion in vivo. Thus, miR-29a may represent a new therapeutic target in glioblastoma.

## Additional file

Additional file 1: **Figure S1.** Effect of miR-29a on Sox4 mRNA. **Figure S2.** miR-29a mimic increases invasion in primary glioblastoma stem-like cells. **Figure S3.** Sox4 is a miR-29a target. **Figure S4.** Effect of a miR-29a sponge on glioblastoma cell growth. **Figure S5.** Effect of a miR-29a sponge on glioblastoma cell invasion. **Figure S6.** Schematic diagram of mechanisms. (PDF 441 kb)

## Abbreviations

GBM: Glioblastoma
GFP: Green fluorescent protein
IACUC: Institutional Animal Care and Use Committee
PCR: Polymerase chain reaction
RFP: Red fluorescent protein
TCGA: The Cancer Genome Atlas
