# Supplementary material for: MicroRNA-29a activates a multi-component growth and invasion program in glioblastoma
Source: J Exp Clin Cancer Res. 2019 Jan 25;38:36. doi: 10.1186/s13046-019-1026-1 (PMC6347789; doi:10.1186/s13046-019-1026-1)
Supplement: Supplementary file 1 — Figure S1. Effect of miR-29a on Sox4 mRNA. Figure S2. miR-29a mimic increases invasion in primary glioblastoma stem-like cells. Figure S3. Sox4 is a miR-29a target. Figure S4. Effect of a miR-29a sponge on glioblastoma cell growth. Figure S5. Effect of a miR-29a sponge on glioblastoma cell invasion. Figure S6. Schematic diagram of mechanisms. (PDF 441 kb) [file 13046_2019_1026_MOESM1_ESM.pdf]

Supplementary Figure S1

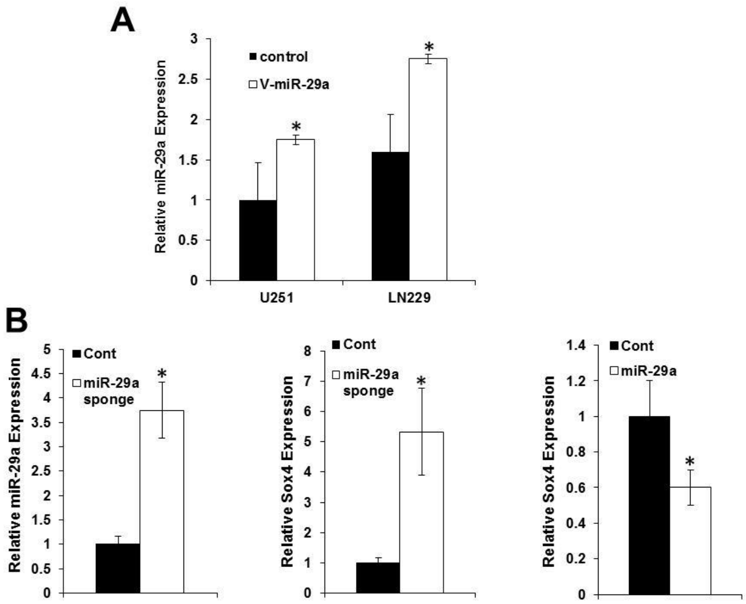

Supplementary Figure S1: ***A)*** Real-time PCR of miR-29a expression in human U251 or LN229 glioblastoma cells transduced with a control lentivirus or a miR-29a lentivirus. Data shown are mean ± SD. *=*P*<0.05, unpaired t-test. ***B)*** Real-time PCR of miR-29a or Sox4 mRNA expression in human U251 glioblastoma cells transduced with a lentivirus encoding either a miR-29a sponge or miR-29a itself. Data shown are mean ± SE. *=*P*<0.05, unpaired t-test.

Supplementary Figure S2

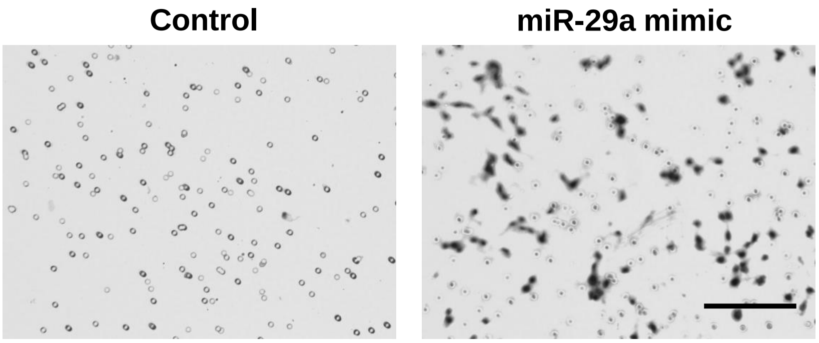

Supplementary Figure S2: Phase microscope images from Matrigel invasion assay illustrating effect of a miR-29a mimic (100 nM) on invasion by primary glioblastoma stem-like cells. Scale is approximately 350 uM.

Supplementary Figure S3

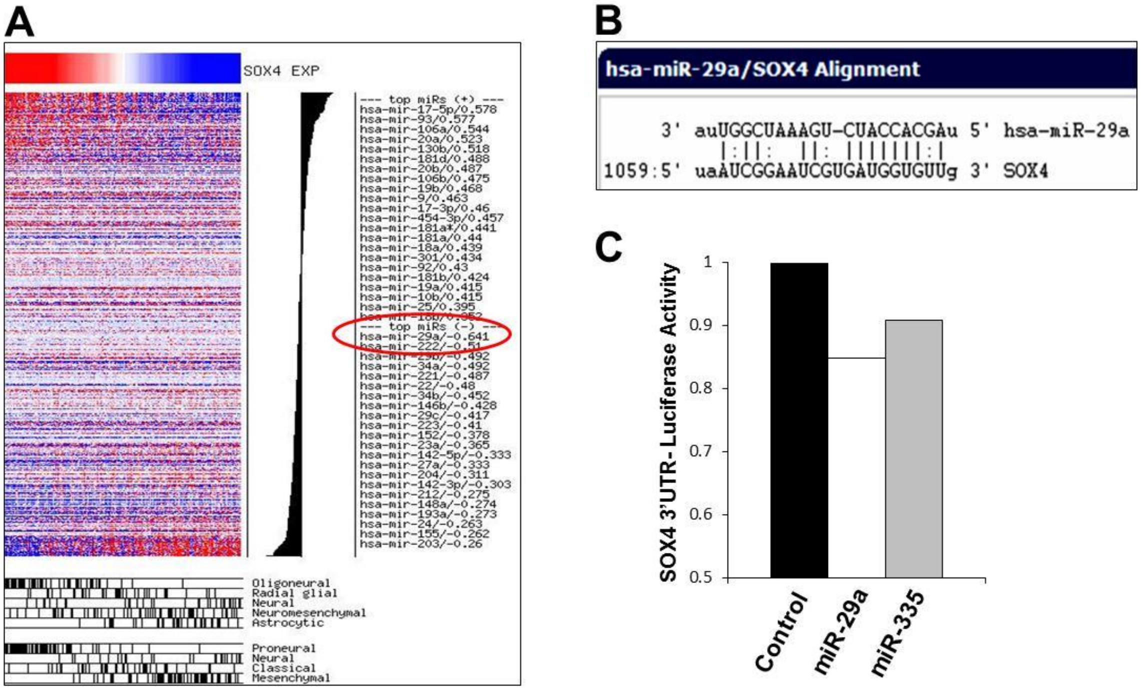

Supplementary Figure S3: Sox4 is a miR-29a target. ***A)*** TCGA mRNA and microRNA expression data from 197 human glioblastoma specimens. The microRNA and mRNA-based subclassification of the individual specimens is indicated at bottom.The microRNAs that are most positively correlated or most negatively correlated with Sox4 mRNA expression are rank ordered and listed to the right. The red circle identifies miR-29a as the most anti-correlated microRNA to Sox4**.** ***B)*** Predicted miR-29a binding site in the 3'-UTR of the Sox4 mRNA. ***C)*** Luciferase assay demonstrating the effect of a miR-29a mimic (100 nM) or a control mimic on the activity of a luciferase-Sox4-3'-UTR fusion protein expressed in human 293T cells. A miR-335 mimic was used as a positive control, as this has been reported to directly target the Sox4 3'-UTR. Data shown is the mean of 4 replicates. $P<0.05$, unpaired t-test.

Supplementary Figure S4

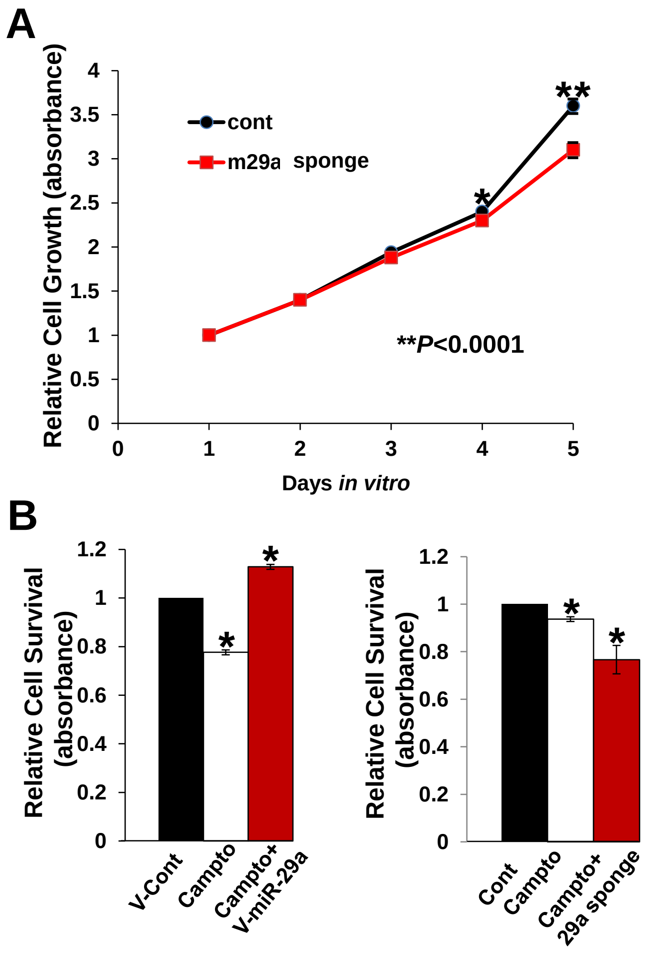

Supplementary Figure S4: Effect of a miR-29a sponge on glioblastoma cell growth. ***A)*** MTT growth assay illustrating the effect of lentiviral-mediated overexpression of a miR-29a sponge (miR-29a locker) or a control sponge on the growth of U251 glioblastoma cells *in vitro*. Data shown are the mean ± SEM of eight replicates. *=*P*<0.05, **=*P*<0.0001, unpaired t-test. B) *left panel* Effect of an 18 hour exposure to camptothecin (Campto, 100 µM) or vehicle alone (control) on the survival of human U251 glioblastoma cells transduced with a control lentivirus or a miR-29a lentivirus. *right panel* Effect of an 18 hour exposure to camptothecin (Campto, 100 µM) or vehicle alone (control) on the survival of human U251 glioblastoma cells transduced with a lentivirus encoding a control microRNA sponge or a miR-29a sponge. *=*P*<0.05, t-test

Supplementary Figure S5

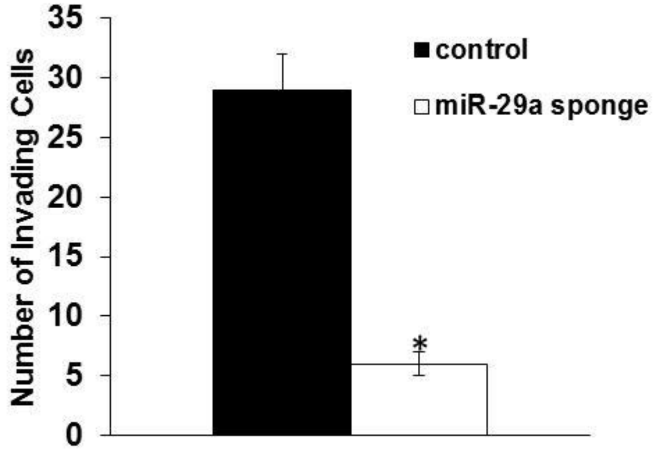

Supplementary Figure S5: Effect of a miR-29a sponge on glioblastoma cell invasion. Matrigel invasion assay illustrating the effect of lentiviral-mediated overexpression of a miR-29a sponge (miR-29a locker) or a control sponge on the invasion of U251 glioblastoma cells *in vitro*. Data shown are mean ± SEM. *=P<0.0001, unpaired t-test.

Supplementary Figure S6

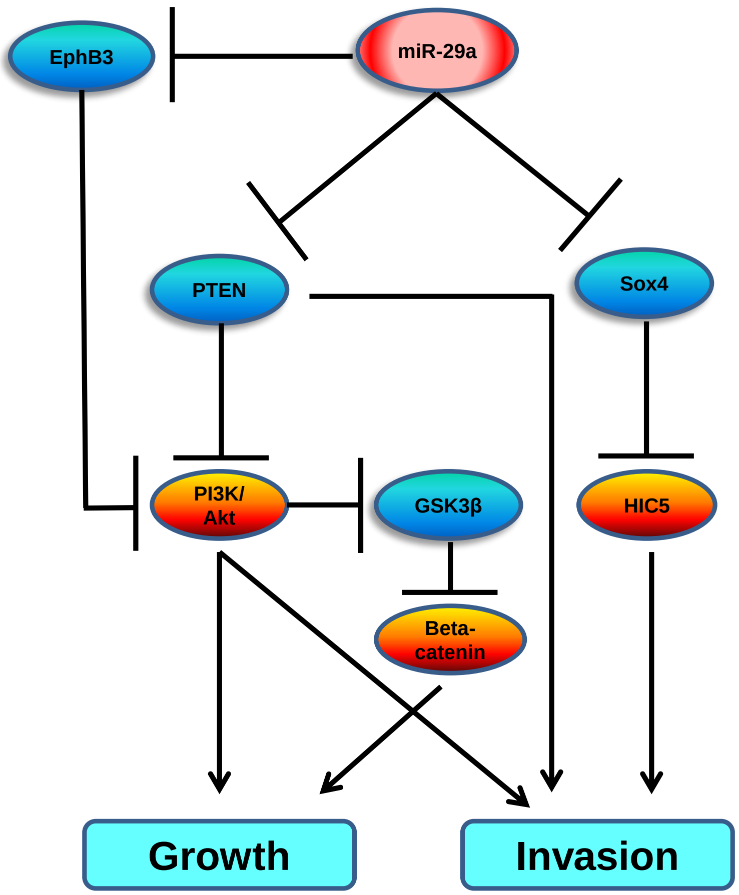

Supplementary Figure S6: Schematic diagram illustrating proposed mechanisms underlying the effects of miR-29a on glioblastoma cell growth and invasion.
